# Inhibition of Histone Methyltransferase, Histone Deacetylase, and *β*-Catenin Synergistically Enhance the Cardiac Potential of Bone Marrow Cells

**DOI:** 10.1155/2017/3464953

**Published:** 2017-07-16

**Authors:** Jinpu Yang, Keerat Kaur, John G. Edwards, Carol A. Eisenberg, Leonard M. Eisenberg

**Affiliations:** ^1^New York Medical College/Westchester Medical Center Stem Cell Laboratory, Departments of Physiology and Medicine, New York Medical College, Valhalla, NY 10595, USA; ^2^Department of Biology and Genomics, New York University, New York, NY 10003, USA; ^3^Department of Physiology, New York Medical College, Valhalla, NY 10595, USA

## Abstract

Previously, we reported that treatment with the G9a histone methyltransferase inhibitor BIX01294 causes bone marrow mesenchymal stem cells (MSCs) to exhibit a cardiocompetent phenotype, as indicated by the induction of the precardiac markers Mesp1 and brachyury. Here, we report that combining the histone deacetylase inhibitor trichostatin A (TSA) with BIX01294 synergistically enhances MSC cardiogenesis. Although TSA by itself had no effect on cardiac gene expression, coaddition of TSA to MSC cultures enhanced BIX01294-induced levels of Mesp1 and brachyury expression 5.6- and 7.2-fold. Moreover, MSCs exposed to the cardiogenic stimulus Wnt11 generated 2.6- to 5.6-fold higher levels of the cardiomyocyte markers GATA4, Nkx2.5, and myocardin when pretreated with TSA in addition to BIX01294. MSC cultures also showed a corresponding increase in the prevalence of sarcomeric protein-positive cells when treated with these small molecule inhibitors. These results correlated with data showing synergism between (1) TSA and BIX01294 in promoting acetylation of lysine 27 on histone H3 and (2) BIX01294 and Wnt11 in decreasing *β*-catenin accumulation in MSCs. The implications of these findings are discussed in light of observations in the early embryo on the importance of *β*-catenin signaling and histone modifications for cardiomyocyte differentiation and heart development.

## 1. Introduction

The prevalence of cardiovascular diseases, and their impact on human health, has spurred considerable research efforts into discovering new therapies. Many potential therapeutic avenues have been explored including novel surgical interventions, genetic manipulations, cytokine injections, and cell transplantations. Great hope was placed on stem cell-based treatments, which have yet to deliver on their initial promise as a panacea for cardiac disease. However, cardiac-competent stem cells are still the focus of therapeutic research, as scientists continue to investigate the capabilities of stem cells to heal the heart either by their introduction into native tissue as nondifferentiated cells or as a source of cells that can be used for generating differentiated myocytes within bioengineered tissue [1–5].

Bone marrow has long been studied as a potential resource for treating the heart due to its accessibility and abundance of stem cells [6–11]. However, initial enthusiasm for using bone marrow to heal the heart has been tempered with disappointment, as stem cells from this noncardiac tissue have not shown a sufficient native potential to generate myocardial tissue [9, 10, 12]. Recently, we reported that exposure of bone marrow stem cells to the G9a histone methyltransferase (HMTase) inhibitor BIX01294 can enhance their cardiac competency [13, 14]. Bone marrow mesenchymal stem cells (MSCs) treated with BIX01294 showed an induced expression of Mesp1 and brachyury [13, 14], which are markers associated with precardiac progenitors in the early embryo [15–17]. Moreover, BIX01294 treatment subsequently allowed MSCs to undergo myocardial differentiation in response to Wnt11 [13, 14], which is a primary extracellular factor that initiates cardiogenesis during early development [18–21].

Wnt proteins are generally classified into two groups, based on the primary signal transduction pathways that they stimulate [22–26]. In the embryo, Wnt11 promotes cardiogenesis via noncanonical Wnt pathways [18–21, 23], which are distinct from canonical Wnt pathways that signal via *β*-catenin [22, 26]. Canonical Wnts, such as Wnt3a, stimulate cellular responses by preventing the degradation of *β*-catenin, which allows this latter protein to accumulate and transduce signal. Although canonical Wnt signaling plays a positive role in advancing the development of the heart after it initially takes form during embryogenesis, inhibition of *β*-catenin-mediated signaling is believed to be a key event initiating cardiac specification in the early embryo [21, 23, 27, 28].

The effect of BIX01294 on bone marrow cells was discovered as part of a large screen of pharmacological reagents for their effect on the cardiopotency of bone marrow stem cells [13, 14]. Drugs used in this analysis were selected based on their previously described utility for assisting the production and/or maintenance of pluripotent stem cells in combination with other treatments [29–32]. In our screening assays, these drugs were tested whether they were capable of broadening the cell phenotypic potential on bone marrow stem cells, without making the cells pluripotent [13, 14]. Of the drugs we analyzed, only BIX01294 displayed a capability to boost the cardiac competency of bone marrow cells. However, since the other drugs we screened had a reported efficacy in helping generate pluripotent cells, but only as part of a compound treatment, we examined whether any of these reagents were capable of further enhancing the effect of BIX01294 in broadening the differentiation potential of bone marrow cells. Here, we report that the histone deacetylase (HDAC) inhibitor trichostatin A (TSA) synergizes with BIX01294 in inducing cardiac gene expression from MSCs. Moreover, analysis of the mechanisms that underlie the acquisition of cardiac competency by MSCs demonstrates that G9a HMTase inhibition displays synergy with HDAC suppression in modifying histone H3 and with Wnt11 in blocking *β*-catenin accumulation within stem cell cultures.

## 2. Material and Methods

### 2.1. Isolation and Culture of Bone Marrow MSCs

The Institutional Animal Care and Use Committee at New York Medical College approved all animal protocols of this study. Bone marrow was harvested from 8–12 wk C57BL/6 mice, as described [33, 34]. MSCs were obtained from bone marrow using standard procedures [35, 36]. Dissociated bone marrow cells were resuspended at 10^6^ cells/ml in Iscove's Modified Dulbecco's Medium (IMDM)/20% FBS and plated onto tissue culture plastic, with nonadherent cells removed after 4 hrs. The remaining adherent cells were cultured for 2 weeks and then split when still subconfluent. Cultures were harvested at the second passage, just prior to reaching confluency, for use in experimentation.

Treatments of MSCs were initiated by serum starvation overnight in IMDM and then culturing in fresh 10% FBS/IMDM in the absence or presence of various doses of BIX01294, 1,5-naphthyridine pyrazole derivative-19 (Npy19; RepSox), CHIR99021, IWP4 (Stemgent), 5-azacytidine (Sigma), 3-bromo-7-nitroindazole, and/or trichostatin A (Cayman Chemical). Two days later, cells were either harvested for RNA or cultured for an additional seven days in fresh 10% FBS/IMDM medium, plus or minus Wnt11, or Wnt3a (250 ng/ml; PeproTech) as previously described [13, 14].

### 2.2. RNA Isolation and PCR Amplification

Quick-RNA MiniPrep kits (Zymo Research) were used to obtain total RNA, which was subsequently reverse-transcribed with Moloney murine leukemia virus reverse transcriptase (Promega). Comparative levels of gene expression were determined by quantitative real-time PCR (qPCR) analysis using the SYBR Green qPCR Master Mix kit (http://bimake.com). Levels of phenotype-specific gene expression were normalized to the expression of the housekeeping gene GAPDH and calculated by the ∆∆Ct method, as described [14, 37].

### 2.3. Immunofluorescence and Immunoblotting

Immunofluorescent labeling of cultured cells was performed as previously described [38, 39]. Mouse anti-*β*-catenin monoclonal antibody (BD Transduction Laboratories) was applied to MSC cultures following processing with Dent's fixative (80% methanol/20% DMSO) and overnight block with 1% BSA/PBS. Staining with antimuscle *α*-actinin (EA-53, Sigma) and titin (9D10, Developmental Studies Hybridoma Bank) antibodies followed formalin fixation and overnight blocking with 5% BSA/PBS. DyLight 488 or TRITC-conjugated secondary antibody (Jackson ImmunoResearch) was used to detect primary antibody labeling of the cultures, with cells counterstained with 4′,6′-diamidino-2-phenyindole (DAPI; Life Technologies) to identify nuclei.

For immunoblot analysis, protein was extracted from MSCs by lysis in RIPA buffer (50 mM Tris-HCl, pH 7.5, 150 mM NaCl, 1% sodium deoxycholate, 0.1% sodium dodecyl sulfate, and 1% Triton X-100) that contained Mammalian ProteaseArrest protease inhibitors (G-Biosciences). Polyacrylamide gel electrophoresis was used to separate total protein, which was transferred to polyvinyl difluoride membrane, and incubated with rabbit antibodies specific to dimethylated H3K9, dimethylated H3K27, acetylated H3K9, acetylated H3K27 (Cell Signaling Technology), or total histone H3 (Millipore). Antibody labeling was detected using alkaline phosphatase-coupled anti-rabbit IgG antibody (Promega) and Luminata Forte Western HRP substrate (EMD Millipore).

### 2.4. Tabulating Immunolabeled Positive Cells

The percentage of MSCs that expressed muscle *α*-actinin or *β*-catenin protein under various culture conditions was determined from imaging immunofluorescent labeled cell cultures. MSCs were plated onto 8-well chamber slides (Nunc) and sequentially cultured in the absence or presence of BIX01294±TSA for 2 days and Wnt for 2 or 7 days, prior to immunofluorescent staining against *α*-actinin or *β*-catenin and then counterstaining with DAPI. Digitized images were obtained with a Zeiss LSM 710 confocal microscope using a 10x objective from three distinct fields within the center, upper-right, and lower-left areas of each well. These images were then imported into ImageJ software (http://imagej.net/), with total cell numbers tabulated from DAPI stained images using the automated particle analysis function and immunolabeled cells manually counted using the Cell Counter plugin (Kurt De Vos, University of Sheffield; kurt.devos@iop.kcl.ac.uk). Each experimental condition was repeated ≥three times and the percentage of cells identified by DAPI labeling that stained positive for either of the two proteins being compiled and statistically analyzed.

### 2.5. Statistics

Statistical analysis was determined with ANOVA followed by the Tukey-Kramer test for comparisons between multiple groups and with the unpaired Student's *t*-test for evaluating differences between individual treatments and specific control groups. Statistical significance was computed with the InStat statistical program (GraphPad Software) and defined as *p* ≤ 0.05, with error bars corresponding to the standard error of the mean.

### 2.6. Cocultures of MSCs with Neonatal Rat Cardiomyocytes

Neonatal rat cardiomyocytes were used for coculture with mouse MSCs. Myocytes were obtained from hearts of one-day-old Wistar rats (Taconic Biosciences, Hudson, NY, USA), as previously described [39]. After their isolation, rat cardiomyocytes were allowed to attach for at least 2 days before their use for MSC coculture experimentation. Mouse MSCs were initially pretreated in the presence or absence of 8 *μ*M BIX01294±50 nM TSA for 48 hrs, prior to their labeling with 20 *μ*M carboxyfluorescein succinimidyl ester (CFSE) vital dye (Thermo Fisher Scientific, Waltham, MA, USA) for 1 hour at 37°C. After extensive washing with PBS, CFSE-labeled mouse MSCs were plated onto beating rat cardiomyocytes at a ratio of 1 : 10. Cocultures were provided with fresh 2% FBS/IMDM medium on alternate days and incubated for two weeks before immunofluorescent staining. For cocultures, red fluorescent TRITC-labeled secondary antibody was used in conjunction with the green fluorescent marker CFSE. However, to avoid confusion with the fluorescence obtained from the MSC only cultures, Adobe Photoshop was used to switch the color channels for cocultures, with the immunolabeled protein appearing green, while dye-marked cells are shown as red fluorescence.

## 3. Results

### 3.1. TSA Synergistically Enhances BIX01294-Mediated Responses of Bone Marrow Cells

**Figure 1:**
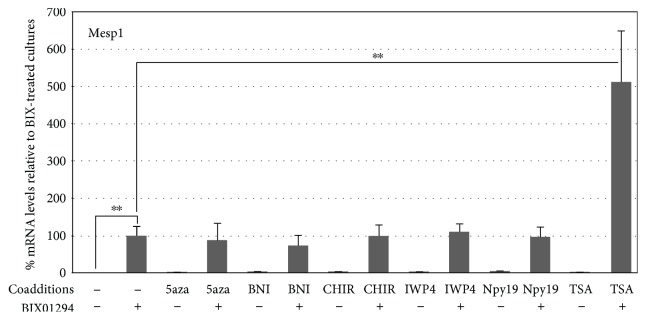
TSA synergistically enhances stimulation of Mesp1 expression by BIX01294. MSCs were incubated with various compounds plus or minus BIX01294 over a broad range of concentrations, with RNA harvested 2 days later and analyzed for Mesp1 expression by qPCR. The chart presents summarized results using optimized doses of inhibitors specific for G9a HMTase (BIX01294), DNA methylation (5-azacytidine (5aza)), nitric oxide synthase (3-bromo-7-nitroindazole (BNI)), GSK3*β* (CHIR99021 (CHIR)), Wnt (IWP4), TGF*β* (1,5-naphthyridine pyrazole derivative-19 (Npy19)), and histone deacetylase (trichostatin A (TSA)). The numbers of biological repeats represented in the graphed data are as follows: control and BIX01294 (*n* = 31) and BIX01294+TSA (*n* = 18), with remaining groups having *n* values ranging from 3 to 5. BIX01294 was the only reagent by itself that was able to induce Mesp1 expression, with >83-fold increase over control levels (^∗∗^*p* < 0.005). None of the other drugs were able to increase Mesp1 expression. However, of these latter drugs, only TSA when combined with BIX01294 produced a significant enhancement of Mesp1-expression over the levels obtained from the BIX01294 only treated cultures (^∗∗^*p* < 0.005). Note that the relative levels of Mesp1 gene expression are normalized to levels obtained from BIX01294-treated MSC cultures.

**Figure 2:**
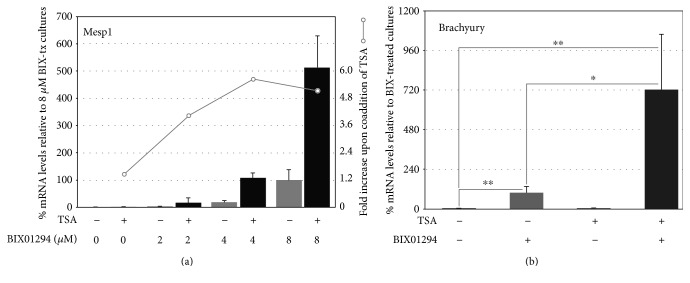
Synergistic enhancement of precardiac gene expression by bone marrow MSCs in response to coaddition of TSA with BIX01294. (a) MSCs were incubated for 48 hrs with various doses of BIX01294 in absence or presence of 50 nM TSA, prior to harvesting RNA and measuring Mesp1 expression by qPCR. On the left side of the chart, the *y*-axis is normalized to Mesp1 levels obtained from MSC cultures treated with 8 *μ*M BIX01294. The right side *y*-axis is scaled to the relative increase in Mesp1 expression resulting from the TSA coaddition to a given concentration of BIX01294. For this series of experiments, BIX01294 generated an ~160-fold increase in Mesp1 expression as compared to control levels, with TSA coaddition enhancing further the Mesp1 response an additional 5.6-fold. The numbers of biological repeats represented in the graphed data are as follows: 2 *μ*M BIX01294 and 2 *μ*M BIX01294+TSA (*n* = 12), with remaining groups having *n* values = 14. (b) RNA was assayed for brachyury expression by qPCR, following its isolation from MSC cultures incubated for 48 hrs in the absence or presence of 8 *μ*M BIX01294 and/or 50 nM TSA. The coaddition of TSA synergistically enhanced BIX01294-stimulation of brachyury expression 7.2-fold (*n* = 14; ^∗^*p* < 0.05; ^∗∗^*p* < 0.01).

In previous reports [13, 14], we described a molecular screen where bone marrow stem cells were exposed to a variety of small molecule inhibitors that had shown utility in assisting the production of induced pluripotent stem cells (iPSCs). When these iPSC helper molecules were tested for their ability to broaden the potential of bone marrow stem cells, the only one that appreciably upregulated precardiac markers and allowed the cells to respond to cardiogenic signals was the G9a HMTase inhibitor BIX01294. As we reported, maximum induction of the precardiac markers Mesp1 and brachyury was obtained in cultures treated for 48 hrs with 8 *μ*M BIX01294. In the present study, we examined if we could increase cardiac gene expression further by employing a secondary screen to identify molecules that could act synergistically with BIX01294 for their effect on bone marrow stem cells. For these experiments, we examined iPSC helper molecules that were unable to promote precardiac gene expression in the initial screen, for their ability to enhance the BIX01294 effect on bone marrow MSCs. Therefore, MSCs were treated with or without 8 *μ*M BIX01294 plus/minus various iPSC helper molecules over a range of concentrations. After 48 hrs, cultures were assayed for induction of the Mesp1 gene, whose expression is regarded as a marker of the precardiac phenotype in the embryo [15, 17]. These new experiments confirmed that none of the iPSC helper molecules, save BIX01294, induced Mesp1 expression (Figure 1). More importantly, none of the other molecules further increased the level of Mesp1 gene expression induced by BIX01294, except the histone deacetylase (HDAC) inhibitor trichostatin A (TSA). Depending on the dosage of these two molecules, TSA was able to enhance the Mesp1 response of MSCs to BIX01294 as much as 5.6-fold (Figures 1 and 2(a)). Overall, the highest levels of Mesp1 expression were generated from MSCs exposed to the combined treatment of 8 *μ*M BIX01294 plus 50 nM TSA for 48 hrs (Figure 2(a)). Similar results were obtained for brachyury gene expression, as TSA was the only molecule tested that demonstrated synergy with BIX01294, with the two drugs generating levels of brachyury expression that were on average 7.2-fold higher than elicited with BIX01294 alone (Figure 2(b)).

### 3.2. BIX01294 and TSA Cooperatively Promote Cardiac Gene Expression by MSCs

**Figure 3:**
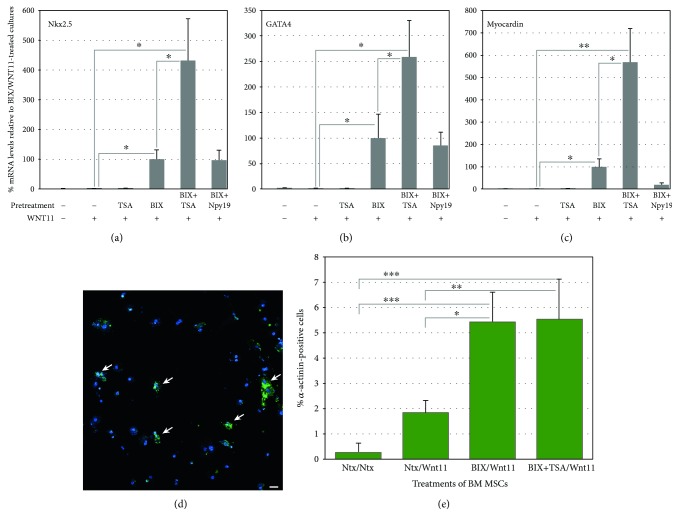
TSA coaddition with BIX01294 promotes greater responsiveness to the cardiogenic stimulating factor Wnt11. MSCs were cultured in the absence or presence of 8 *μ*M BIX01294 plus or minus either 50 nM TSA or 1 *μ*M Npy19 for 48 hrs, prior to seven-day culture in fresh media with or without Wnt11. (a)–(c) Real-time qPCR analysis of RNA harvested from the cultures indicates that the primary cardiac transcription factors Nkx2.5, GATA4, and myocardin were significantly upregulated in MSCs in response to Wnt11, but only when pretreated with BIX01294. When TSA (but not Npy19) was combined with BIX01294 during the initial 48 hr incubation period, a significant enhancement was observed for Nkx2.5 (*n* = 11), GATA4 (*n* = 11), and myocardin (*n* = 8) of 4.3-, 2.6-, and 5.6-fold, respectively. Statistical significance is shown for comparative levels of Nkx2.5, GATA4, or myocardin expression displayed by Wnt11 stimulated cultures pretreated with either TSA plus BIX01294 or BIX01294, and nonpretreatment controls (^∗^*p* < 0.05; ^∗∗^*p* < 0.01). (d) Identification of individual sarcomeric *α*-actinin positive cells (arrows) by immunofluorescent staining indicated that cardiac protein expression within the MSC cultures was in accordance to the gene expression patterns, as shown in this panel for cultures treated sequentially with TSA plus BIX01294 followed by Wnt11. Scale bar = 25 *μ*m. (e) Tabulation of *α*-actinin positive cells within these cultures demonstrated that BIX01294 or BIX01294+TSA pretreatments significantly increased the number of cardiac protein expressing cells as compared to nontreated or Wnt11 only conditions. Statistical significance is indicated by ^∗^*p* < 0.05; ^∗∗^*p* < 0.01; ^∗∗∗^*p* < 0.001.

The above results indicated that TSA synergistically enhanced the induced expression by BIX01294 of key transcription factors characteristic of embryonic precardiac progenitors (Figures 1 and 2). Subsequent experimentation demonstrated that TSA also displayed synergy with BIX01294 in allowing MSCs to respond to cardiac stimuli and undergo myocardiogenic differentiation (Figure 3). For these latter experiments, MSCs were cultured in the absence or presence of 8 *μ*M BIX01294±TSA for 48 hrs, prior to subsequent incubation with the cardiogenic stimulating factor Wnt11. After 7 days of culture with Wnt11, RNA was harvested from the cells and analyzed for cardiac gene expression by real-time qPCR. MSCs collected from cultures that were not exposed to BIX01294 displayed only minimal expression of cardiac genes, regardless of whether or not they were subsequently treated with Wnt11. However, preincubation of MSCs with BIX01294 allowed for the induced expression of the cardiac transcription factors Nkx2.5, GATA4, and myocardin in response to Wnt11 (Figures 3(a), 3(b), and 3(c)). No enhancement over this BIX01294-mediated response was observed when the cultures were supplemented during the 48 hr pretreatment phase with other iPSC helper molecules (e.g., the TGF*β* inhibitor Npy19)—except when TSA was supplied. Again, BIX01294 and TSA displayed synergy, as this combined pretreatment allowed Wnt11 to generate levels of Nkx2.5, GATA4, and myocardin that were 4.3-, 2.6-, and 5.6-fold greater than those produced by cultures that were pretreated with BIX01294 alone (Figures 3(a), 3(b), and 3(c)).

**Figure 4:**
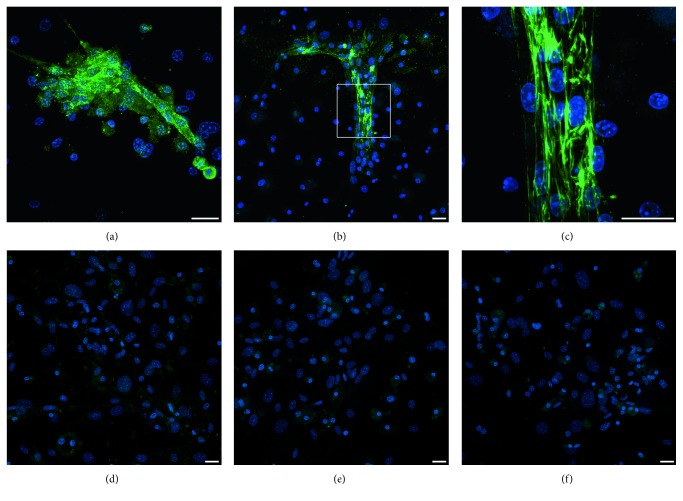
Expression of sarcomeric *α*-actinin by MSCs cultured under various conditions. MSCs were immunostained for *α*-actinin (green) and DAPI counterstained (blue), after incubation in the presence or absence of BIX01294 and/or TSA for 2 days and Wnt treatments for 7 days. MSCs treated with Wnt11 following exposure to (a) BIX01294 or (b), (c) BIX01294+TSA exhibited clusters of *α*-actinin-positive cells. (c) A higher magnification view of the boxed area in the previous panel indicates that the high intensity *α*-actinin staining was not yet exhibited in a striated pattern. In contrast, *α*-actinin staining was not observed either if the cultures were (d) not pretreated with BIX01294±TSA or if pretreatment with BIX01294 and TSA was followed by either (e) the absence of Wnt11 or (f) treatment with Wnt3a instead of Wnt11. Scale bar = 25 *μ*m.

**Figure 5:**
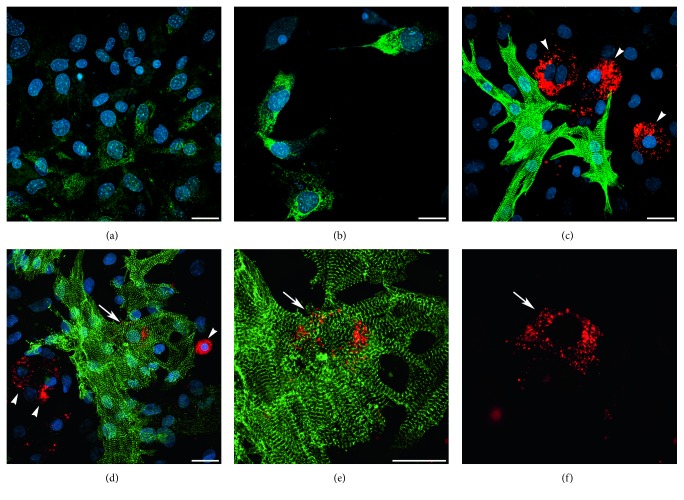
Cardiac differentiation of MSCs following treatment with BIX01294±TSA. (a), (b) MSCs were immunostained for titin (green) and DAPI counterstained (blue), after 7 day incubation with Wnt11, following pretreatment with (a) BIX01294 or (b) BIX01294+TSA. (c)–(f) Coculture of CFSE vital dye-labeled MSCs (red) with neonatal rat cardiomyocytes that were immunostained for *α*-actinin (green) and DAPI counterstained (blue). (c) MSCs that were nontreated prior to coincubation with rat myocytes did not display cardiac phenotypes, as indicated by cells that exhibited only the red vital dye (arrowheads). (d)–(f) In contrast, MSCs pretreated with BIX01294 label showed evidence of cardiac differentiation when in proximity to the rat myocytes. (d) While the majority of MSCs in these later cultures remained nondifferentiated, as indicated by the sole display of the red vital dye (arrowheads), individual BIX01294-treated MSCs were observed that exhibited dual red and green fluorescence (arrow). (e), (f) Higher-magnification view of this coculture shown in the successive panels for both the vital dye fluorescence and actinin immunostaining or vital dye-label only indicates that the red and green colabeled MSC-derived cell (arrow) exhibits *α*-actinin in a striated pattern. Scale bar = 25 *μ*m.

We next examined the extent that BIX01294 and TSA enhanced the prevalence of cardiac phenotypes within the MSC-derived cultures. For these experiments, MSCs were plated at densities that allowed individual cardiac protein positive cells to be definitively identified within the cultures. MSCs were sequentially incubated in the absence or presence of BIX01294±TSA for 2 days and Wnt11 for 7 days, prior to immunofluorescent staining for sarcomeric *α*-actinin (Figure 3(d)). Tabulation of immunofluorescent-labeled cells (Figure 3(e)) indicated that Wnt11 in combination with BIX01294 or BIX01294+TSA pretreatments exhibited *α*-actinin staining in 5.46 and 5.57% of cells, respectively, within the cultures as compared to cells cultured with Wnt11 only (1.87%) or nontreated conditions (0.32%). MSCs plated at higher densities generated clusters of *α*-actinin-positive cells in response to Wnt11 when pre-exposed to BIX01294 (Figure 4(a)) or BIX01294+TSA (Figures 4(b) and 4(c)). High-resolution views of these *α*-actinin-positive cells within these cultures indicated that this sarcomeric protein was exhibited in a nonstriated pattern (Figure 4(c)). In contrast, *α*-actinin-positive cell clusters were not observed if the cultures were either not pretreated with BIX01294±TSA (Figure 4(d)), or if Wnt11 was absent (Figure 4(e)) or replaced with the noncardiogenic growth factor Wnt3a (Figure 4(f)) following pretreatment with BIX01294±TSA. Further indications that MSCs could be converted to cardiac phenotypes was provided by their expression of the sarcomeric protein titin when stimulated with Wnt11 after exposure to BIX01294 (Figure 5(a)) or BIX01294+TSA (Figure 5(b)). Consistent with these results were experimental data indicating that BIX01294 pretreatment enabled MSCs to undergo cardiac differentiation and show evidence of a striated muscle phenotype when cocultured with primary neonatal rat cardiomyocytes (Figures 5(c), 5(d), 5(e), and 5(f)).

### 3.3. Mechanisms of BIX01294 and TSA Action on Bone Marrow MSCs

**Figure 6:**
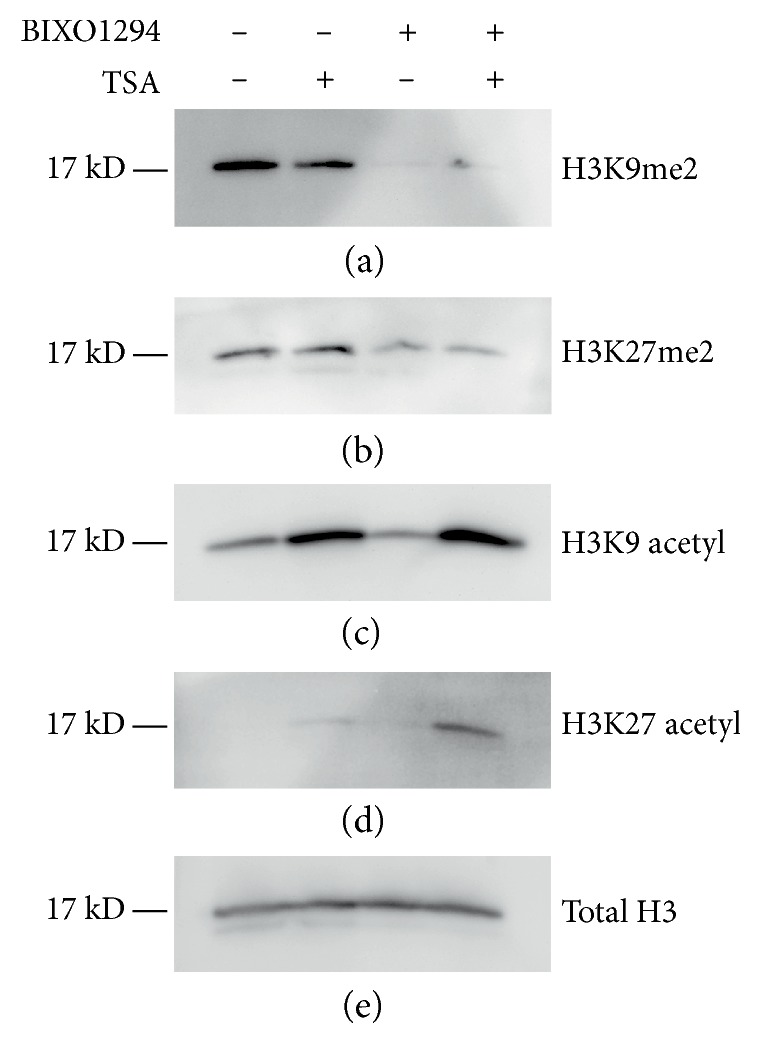
Modification of histone H3 by BIX01294 and TSA. Protein was isolated from bone marrow MSCs that were cultured in the absence or presence of BIX01294 and/or TSA. Following electrophoretic separation, the protein preparations were blotted with antibodies that recognized either total or specifically modified histone H3. (a), (b) Immunoblots of total protein demonstrated that methylation of H3K9 and H3K27 was reduced by G9a HMTase inhibition by BIX01294. (c) Treatment with the HDAC inhibitor TSA led to increased acetylation at H3K9. (d) While culturing MSCs with TSA also enhanced H3K27 acetylation, this increase was slight. However, the coaddition of BIX01294 to TSA synergistically stimulated the acetylation of this lysine residue. (e) Blotting for total histone H3 as control verified that each cellular sample consisted of equal amounts of protein.

To begin to decipher how BIX01294 and TSA synergistically upregulated cardiac gene expression, we looked at the global methylation and acetylation patterns of histone H3 (Figure 6). Incubation of MSCs in BIX01294 reduced methylation of histone H3 at both lysine 9 (H3K9) and lysine 27 (H3K27). The coaddition of TSA did not affect this BIX01294-mediated decrease in methylation at H3K9 and H3K27, nor did the presence of TSA by itself reduce histone H3 methylation at either lysine residue (Figures 6(a) and 6(b)). As expected, acetylation at H3K9 and H3K27 was enhanced by TSA, but not by BIX01294. When TSA and BIX01294 were added together, the acetylation at lysine 9 was similar to the levels obtained when TSA was added alone (Figure 6(c)). Yet, the acetylation pattern at lysine 27 had a different result, as coaddition of BIX01294 synergistically enhanced the TSA-mediated acetylation at H3K27 (Figure 6(d)), which is a marker of active enhancers that are enriched during cardiac development [40–43].

**Figure 7:**
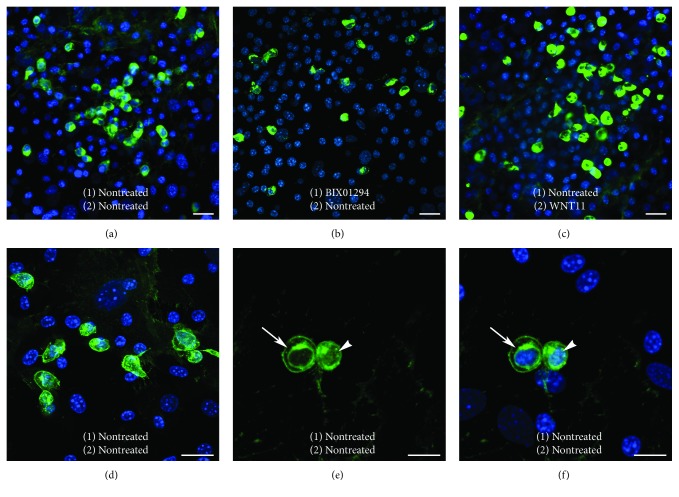
*β*-catenin expression within MSCs cultures. MSCs were immunostained for *β*-catenin (green) and nuclear counterstained with DAPI (blue), following a two-step protocol that involved the culturing of cells (1) for 2 days in the absence or presence of BIX01294 and then (2) for an additional 2 days with or without Wnt11. (a) MSCs cultured without treatment contained many brightly stained *β*-catenin-positive cells. (b) Cultures treated with BIX01294 without Wnt11 or (c) with Wnt11 without pretreatment also displayed many brightly stained *β*-catenin-positive cells. (d)–(f) Higher magnification views of nontreated MSCs revealed the pattern of *β*-catenin fluorescence within these cells. (e), (f) High resolution of brightly stained *β*-catenin-positive cells shown in successive panels for *β*-catenin immunoreactivity only or both *β*-catenin and DAPI fluorescence, indicated that *β*-catenin protein is displayed within the cytoplasm, perinuclear region (arrow), and nucleus (arrow). Scale bar = 20 *μ*m.

**Figure 8:**
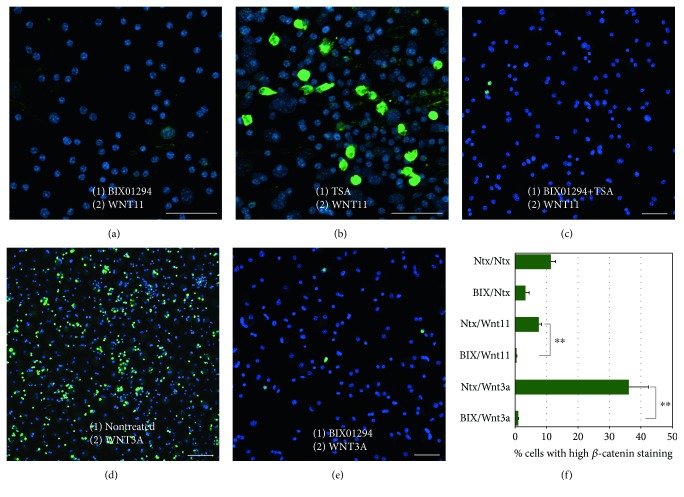
Differential *β*-catenin immunoreactivity by MSCs cultured under various conditions. MSCs were fluorescently labeled with *β*-catenin antibody (green) and DAPI nuclear stain (blue), after culturing cells for 2 days in the absence or presence of BIX01294 and/or TSA and for an additional 2 days with or without Wnt11 or Wnt3a. (a) MSCs cultured in sequence with BIX01294 and Wnt11 were deficient in cells that brightly stained for *β*-catenin. In contrast, (b) cultures treated sequentially with TSA and Wnt11 still displayed numerous brightly stained *β*-catenin-positive cells. (c) However, MSCs pretreated with both BIX01294 and TSA, followed by Wnt11 exposure, no longer displayed cells with high-intensity *β*-catenin immunoreactivity. (d) MSCs treated with Wnt3a showed a marked increase in *β*-catenin-positive cells. (e) Yet, this high-intensity *β*-catenin immunoreactivity disappeared when MSCs were first exposed to BIX01294 prior to treatment with Wnt3a. Scale bar = 50 *μ*m. (f) Tabulation of immunofluorescent cells under these various conditions verified that BIX01294 pretreatments decreased the number of brightly stained *β*-catenin-positive cells that were derived from the cultures. Statistical significance is indicated by ^∗∗^*p* < 0.01.

As a second element in elucidating the regulatory mechanism of BIX01294 and TSA enhancement of MSC cardiac competency, we investigated how these reagents affected the intracellular expression of *β*-catenin, which is a downstream target for Wnt signal transduction. Again, bone marrow MSCs were subjected to a two-part culture protocol with an initial treatment in the absence or presence of BIX01294 plus or minus TSA for 2 days. Subsequently, these MSC cultures were incubated for two additional days with or without Wnt11, prior to their fixation and immunofluorescent staining for *β*-catenin (Figures 7 and 8). Cultures of nontreated MSCs exhibited large numbers of cells that exhibited high-intensity *β*-catenin staining (Figure 7(a)). Prevalence of *β*-catenin-positive cells was not overtly diminished by treatment with BIX01294 (Figure 7(b)). Cultures that were exposed to Wnt11 without prior exposure to BIX01294, also exhibited prominent *β*-catenin staining (Figure 7(c)). When viewed at higher magnification (Figures 7(d), 7(e), and 7(f)), the cells that displayed high *β*-catenin immunoreactivity appeared to exhibit this protein within the cytoplasm, perinuclear region, and nuclei (Figures 7(e) and 7(f)). However, the presence of the brightly stained *β*-catenin-positive cells was eliminated when MSCs were sequentially exposed to BIX01294 and Wnt11 (Figure 8(a)). In contrast, pretreatment with TSA, prior to Wnt11 exposure, did not cause a reduction in *β*-catenin immunoreactivity (Figure 8(b)). But when TSA was combined with BIX01294, subsequent treatment with Wnt11 prevented the appearance of brightly stained *β*-catenin-positive cells (Figure 8(c)). In consideration of the known differential properties of Wnt11 and Wnt3a, it was not surprising that the prevalence of *β*-catenin-positive MSCs increased when cultures were treated with Wnt3a without prior exposure to BIX01294 (Figure 8(d)). What was surprising, however, was the obvious decrease in *β*-catenin immunoreactivity when the cultures were first treated with BIX01294 prior to Wnt3a exposure (Figure 8(e)). This effect of BIX01294 pretreatments in reducing *β*-catenin expression was verified statistically by tallying the numbers of immunoreactive cells that were present under these various conditions. For cultures that were not pretreated with BIX01294, 7.5% and 36% of the cells, respectively, exhibited high *β*-catenin immunoreactivity in response to Wnt11 or Wnt3a. However, cultures treated with either Wnt protein saw a drop in the prevalence of these immunolabeled cells to <1%, when previously exposed to BIX01294. The implications of these findings in regard to crosstalk between epigenetic and WNT regulation of stem cell specification are discussed below.

## 4. Discussion

Bone marrow has long attracted the attention of research scientists and clinicians for use in cardiac repair because it provides an accessible and abundant source of stem cells [6–11]. However, the initial hope that bone marrow stem cells would provide an effective treatment for cardiac disease has yet to come to fruition. The development of technologies for converting adult somatic cells to iPSCs, which in turn can give rise to cardiomyocytes, has indicated that noncardiac tissues may serve as a resource of cells that potentially could be used for repairing a diseased or damaged heart [44–47]. An obstacle in using iPSCs for stem cell therapy is that pluripotent cells possess a proliferative and differentiation potential that is difficult to control and can lead to tumorigenesis [48, 49]. Even when iPSCs are used to generate nonpluripotent progenitor of differentiated cells, or if somatic cells are coaxed to dedifferentiate to a pluripotent state and then subsequently differentiate in the same dish [44], the presence of contaminating pluripotent cells within the final cell preparation may potentially carry a risk of tumor formation or inappropriate tissue remodeling. An alternative approach for producing cardiopotent stem cells from accessible adult tissues is to harvest their stem cells and expose them to treatments that would broaden their differential potential, but without making the cells pluripotent [13, 14]. It is this approach that we have pursued in screening pharmacological reagents, which had been utilized as components of treatments that are able to contribute to generating iPSCs [29–32], for their utility in expanding the differentiation potential of bone marrow MSCs.

The present study is a continuation of our previous efforts into developing protocols for converting MSCs to a cardiac competent phenotype [13, 14]. The hypothesis underlying this research is based on the shared mesodermal lineage of bone marrow and heart. Accordingly, if the differentiation potential of bone marrow MSCs could be broadened, while stopping short of making the cells pluripotent, these cells could be converted to cardiocompetent, panmesodermal cells. Our previous investigations have demonstrated that the G9a HMTase inhibitor BIX01294 will cause these cells to exhibit the early mesodermal, precardiac markers Mesp1 and brachyury, without any corresponding enhancement of either endodermal, ectodermal, or pluripotency markers [13]. Furthermore, we showed that BIX01294 treatments allowed bone marrow cells to express cardiac genes and proteins in response to the cardiogenic stimulus Wnt11 [13, 14]. Here, we report our results with a secondary screen that demonstrated that coaddition of the HDAC inhibitor TSA with BIX01294 increased the expression of Mesp1 and brachyury, 5.6- and 7.2-fold, respectively, over levels induced by BIX01294 exposure alone. Additionally, we have shown that the TSA acted synergistically with BIX01294 to upregulate GATA4, Nkx2.5, and myocardin in response to Wnt11. Thus, incubation of MSCs with Wnt11, following exposure to both BIX01294 and TSA, resulted in GATA4, Nkx2.5, and myocardin expression levels that were 2.6- to 5.6-fold greater than those of cultures pretreated with BIX01294 only—which was 35- to 45-fold above and beyond the levels generated from cultures incubated with Wnt11 without any pretreatment. Moreover, data in this report indicated that these pharmaceutical reagents, which prevent specific modifications of histone H3, significantly enhanced the ability of Wnt11 to promote cardiac protein expression, suggesting that the MSC cultures gave rise to cells exhibiting an immature cardiac phenotype.

**Figure 9:**
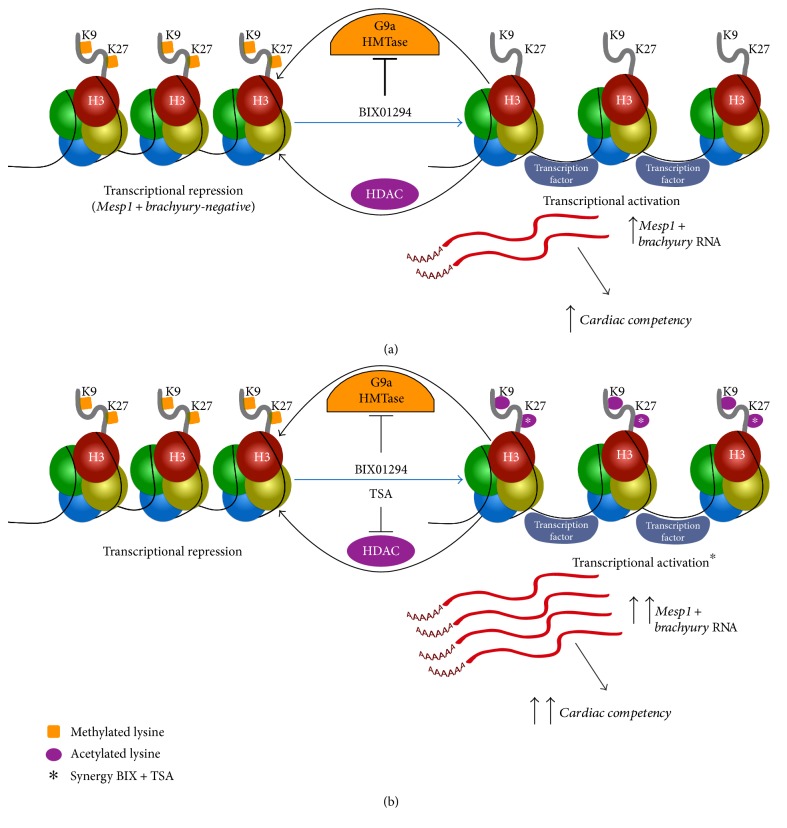
Model for BIX01294 and TSA mediated induction of MSC cardiocompetency. (a) G9a HMTase and HDAC activity in MSCs maintains precardiac genes in a closed chromatin configuration and thus are transcriptionally silent. BIX01294 treatment inhibits G9a HMTase methylation of histone H3 lysine residues H3K9 and H3K27 within genes associated with precardiac progenitors, such as Mesp1 and brachyury, thus allowing these genes to be expressed and conferring MSCs with a precardiac cell potential. (b) While inhibition of HDACs by TSA is not sufficient to promote Mesp1 or brachyury expression from bone marrow MSCs, this drug acts synergistically (asterisk) with BIX01294 in the transcriptional activation of precardiac gene expression and acquisition of a cardiac competent cell phenotype. We hypothesize that this enhanced precardiac gene expression and cardiocompetency supported by the coaddition of these two drugs is due to their cooperative enhancement of H3K27 acetylation (asterisks), which is a known epigenetic indicator of cardiac gene expression in the early embryo.

**Figure 10:**
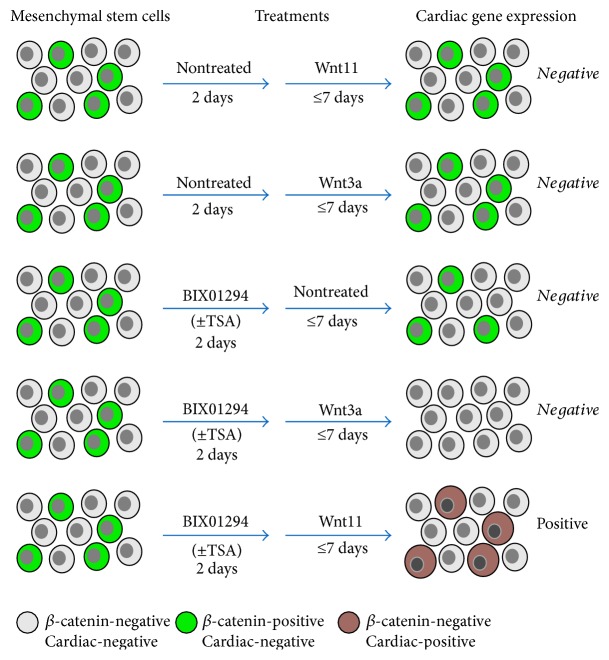
Model of epigenetic and Wnt regulation of cardiogenesis. Results presented in this study indicate that BIX01294 induces a precardiac phenotype from MSCs and acts cooperatively with Wnt11 in promoting both cardiac differentiation and inhibiting the intracellular accumulation of *β*-catenin. Inhibition of *β*-catenin accumulation by cells of the early mesoderm has been postulated to be a key element in the initiation of heart formation. Thus, we hypothesize that the BIX01294 prerequisite for generating cardiac phenotypes from MSCs is twofold: (1) to promote gene expression that is in accordance with a precardiac phenotype and (2) cooperatively allow Wnt11 to reduce cellular *β*-catenin levels, thereby initiating cardiac differentiation of the treated MSCs.

BIX01294 and TSA are small molecule inhibitors of epigenetic modifying enzymes that, respectively, demethylate and acetylate histone lysine residues [50–54]. The mechanistic importance of these epigenetic changes in influencing MSC gene expression and differentiation potential is indicated by two findings outlined in this study, which are summarized in Figures 9 and 10. The first finding, which may explain how the coaddition of TSA boosts BIX01294-mediated cardiac gene expression, is that these two molecules synergistically act to upregulate H3K27 acetylation (Figures 6 and 9). The epigenetic repercussions of BIX01294 and TSA treatments share similarities, as their effect on histone H3 demethylation and acetylation relaxes the structure of chromatin that then promotes gene transcription [55, 56]. Yet, one of the differences between BIX01294 and TSA is that the former is a much more selective inhibitor. BIX01294 has a narrow target range, as its inhibitory activity is specific for the related histone methyltransferases G9a and G9a-related protein (GLP) [50, 52, 57]. Both enzymes act on the same lysine residues of histone H3 and exert their function in vivo by forming homomeric or heteromeric complexes. In contrast, TSA is a broad-spectrum inhibitor that affects all class I, II, and IV HDACs [51, 54, 58]. These latter enzymes catalyze the deacetylation of lysine residues within the aminoterminal tail of core histone proteins H2A, H2B, H3, and H4. In addition, each HDAC protein has multiple nonhistone targets, which may account for the functional differences among the various HDAC proteins and complicate the analysis of HDAC involvement in epigenetic regulation. It is unclear if improved outcomes in promoting a cardiac phenotype could be obtained if more selective HDAC inhibitors were employed. In that regard, experiments using MOCPAC or BATCP—which are preferential inhibitors of class I or II HDACs, respectively [59]—generated results similar to TSA in enhancing BIX01294 stimulation of precardiac gene expression (not shown). This latter observation is supportive of the interpretation that the TSA effect on MSC cultures is due to its influence on histone modification. Moreover, the synergism between BIX01294 and TSA in both promoting cardiac gene expression and H3K27 acetylation correlates with observations in the early embryo that have shown that acetylated H3K27 is a key marker of cardiomyocyte differentiation and heart development [40–43].

The second finding is that BIX01294 pre-exposure acts synergistically with Wnt11 to downregulate cellular *β*-catenin levels. Wnts play a multifaceted role during the development of the heart [21, 23, 27, 60, 61]. Wnt11 is a primary stimulus for heart development and whose expression correlates with a corresponding inhibition of *β*-catenin signaling in the early mesoderm. After the initial formation of the primary heart tube, localized and temporal expression of both canonical and noncanonical Wnts regulate the remodeling and growth of the heart. A hallmark of canonical Wnt signal transduction is that it promotes the stability and activity of *β*-catenin [22–26]. Noncanonical Wnts, such as Wnt11, are thought to act in opposition to canonical Wnt signaling, in part by preventing the intercellular accumulation of *β*-catenin [62–64]. As described in this study, MSC cultures contained large numbers of brightly stained *β*-catenin-positive cells. This high-intensity staining for *β*-catenin persisted in the cultures regardless of whether BIX01294, TSA, or Wnt11 was present. Consistent with the current understanding of Wnt signal transduction, the prevalence of brightly stained *β*-catenin-positive cells increased further when MSCs cultures were treated with the canonical Wnt protein Wnt3a. Surprisingly, there was a significant reduction in the number of cells exhibiting high-intensity *β*-catenin immunoreactivity when cultures were pretreated with BIX01294 and then subsequently exposed to Wnts—regardless of whether the stimulus was a canonical (Wnt3a) or noncanonical (Wnt11) Wnt. Thus, an important signal transduction outcome of Wnt signaling, in regard to *β*-catenin stability, appears to be influenced by the activity of a key epigenetic modulation enzyme G9a HMTase.

The important outcome of our studies is that pretreatment of MSCs with BIX01294±TSA significantly enhanced cardiac gene and protein expression. Although, there appeared to be a correspondence between enhanced cardiogenesis and decreased *β*-catenin, we are unable presently to determine whether the cells whose *β*-catenin expression was downregulated in response to BIX01294 plus Wnt11 are the cells that become cardiac competent in response to these treatments. However, what we can say is that the coincidence of downregulated *β*-catenin expression and increased cardiocompetence within the cultures (summarized in Figure 10) corresponds to the occurrence of these events during cardiac specification in the early embryo. Yet, the observation that BIX01294 pretreatment caused a reduction in *β*-catenin immunoreactivity, but not cardiac protein expression in response to Wnt3a, suggests that there are additional transduction events initiated by Wnt11 for stimulating cardiac differentiation.

In summary, we report that coaddition of the HDAC inhibitor TSA synergistically enhances the ability of the G9a HMTase inhibitor BIX01294 to promote bone marrow MSC cardiocompetency. This observation was demonstrated both by the elevated expression of the embryonic precardiac markers Mesp1 and brachyury and increases in cardiac gene and protein expression following subsequent stimulation of the cultures with Wnt11. The cardiac protein-positive cells described in this report suggest that MSCs may have differentiated to an immature myocardial phenotype. Pretreatment with BIX01294 allowed for Wnt11-mediated reduction of internal *β*-catenin expression and coaddition of TSA with BIX01294 synergistically enhanced H3K27 acetylation—which are both known regulatory events associated with cardiac specification and heart formation in the early embryo. Collectively, these data provide supportive evidence that pharmacological regulation of stem cell phenotype and differentiation potential has utility as an experimental approach for generating cellular tools for the use in cardiac repair.
